# Suzetrigine as a Novel Non-opioid Analgesic Drug in Pain Management: A Review of Clinical Evidence and Therapeutic Perspectives

**DOI:** 10.7759/cureus.90755

**Published:** 2025-08-22

**Authors:** Maciej Mach, Aleksandra Giba, Milosz Miedziaszczyk, Adrian Bryla, Danuta Szkutnik-Fiedler

**Affiliations:** 1 Department of General, Vascular, Endocrine, and Transplant Surgery, Medical University of Warsaw, Warsaw, POL; 2 Department of Clinical Pharmacy and Biopharmacy, Poznań University of Medical Sciences, Poznań, POL; 3 Department of Clinical Pharmacology, Jagiellonian University, Kraków, POL; 4 Department of Clinical Pharmacy, Ludwik Rydygier Specialist Hospital, Kraków, POL

**Keywords:** neuropathic pain, nociceptive pain, non-opioid drug, pain management, suzetrigine

## Abstract

The NaV1.8 sodium channel plays a key role in the transmission of pain signals in peripheral sensory neurons. Suzetrigine is a selective NaV1.8 inhibitor developed as a non-opioid analgesic. Its action is limited to sensory neurons, reducing the risk of adverse effects associated with non-selective sodium channel blockers. Phase II and III clinical trials have demonstrated the high efficacy of suzetrigine in managing postoperative pain, along with good tolerability. Adverse events, such as dizziness or drowsiness, were rare and generally mild to moderate in intensity. The drug did not cause respiratory depression or addiction, which distinguishes it from opioids. Ongoing pharmacokinetic studies and long-term observations aim to further define the drug’s safety profile and its potential use in patients with comorbid conditions. Suzetrigine may offer an effective and safe alternative to opioids in pain treatment.

## Introduction and background

Pain is a subjective, unpleasant sensory and emotional experience associated with actual or potential tissue damage [1]. The process of pain signal transmission can be divided into four main stages: transduction, transmission, modulation, and perception. During transduction, nociceptors detect harmful stimuli and convert them into nerve impulses. These signals are then transmitted to the spinal cord via Aδ and C fibers, and from there, they ascend through the spinothalamic tract to the thalamus [2].

At the transmission stage, pain signals may undergo modification through modulatory processes that influence individual pain perception [3]. Modulation occurs at various levels of the nervous system and includes mechanisms such as the activation of opioid receptors by endogenous and exogenous opioids, autonomic responses, and regulation of N-methyl-D-aspartate (NMDA) receptor and gamma-aminobutyric acid (GABA) signaling [3].

Once the signal reaches the brain, perception occurs - a process involving multiple cortical and subcortical structures that not only interpret the pain stimuli but also dynamically modulate them [2]. Analgesic drugs act at various stages of the pain pathway to reduce its intensity.

Despite being a cornerstone of moderate to severe pain management, opioids present significant limitations, including the risk of tolerance, dependence, respiratory depression, and opioid-induced hyperalgesia [4]. The ongoing opioid crisis has further highlighted the urgent need for alternative analgesics that provide effective pain relief without these adverse outcomes.

**Figure 1:**
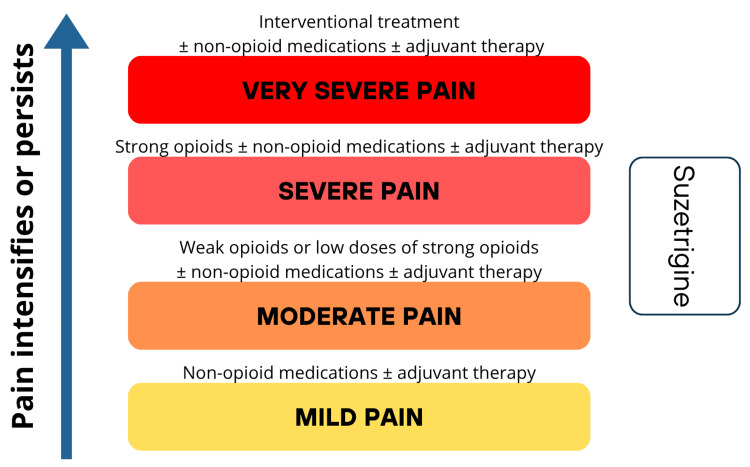
The WHO analgesic ladder with the proposed positioning of suzetrigine Image credit: Maciej Mach

Depending on the type of pain, non-opioid treatments may include first-step analgesics (according to the analgesic ladder), as well as non-selective sodium channel blockers (e.g., lidocaine, mexiletine, and carbamazepine), gabapentinoids, and antidepressants [5]. The World Health Organization (WHO) analgesic ladder is a stepwise approach to pain management, illustrated in Figure 1 with an example placement of suzetrigine.

Non-steroidal anti-inflammatory drugs (NSAIDs) and paracetamol relieve pain by inhibiting enzymes responsible for inflammatory processes - cyclooxygenase (COX) and peroxidase (POX). Local anesthetics block the conduction of pain impulses by inhibiting sodium channels, whereas gabapentinoids affect glutamate uptake and block calcium channels. NMDA receptor antagonists, such as ketamine, as well as opioids, contribute to the modulation of pain pathways, thereby reducing the sensation of pain [3].

In pain management, it is essential to maintain a balance between providing adequate analgesia and minimizing the risk of adverse effects. Recently, researchers have discovered that NaV1.8 - a specific sodium channel in the body - plays a key role in how pain is perceived. Drugs that block NaV1.8, such as suzetrigine, may offer effective pain relief. Suzetrigine is an oral, selective NaV1.8 inhibitor that has demonstrated more than 31,000-fold greater selectivity for NaV1.8 compared to other NaV subtypes [6].

The goal of this article is to evaluate the therapeutic potential and safety profile of suzetrigine, employing a systematic review of preclinical and clinical studies as the primary method to synthesize current evidence.

## Review

Mechanism of action

The discovery of the role that voltage-gated sodium channels (NaV) play in pain transmission has initiated research into the potential analgesic applications of NaV modulators. Among the nine known NaV subtypes (NaV1.1 to NaV1.9), NaV1.7, NaV1.8, and NaV1.9 are preferentially expressed in nociceptive neurons within the peripheral nervous system [7].

Through this mechanism, the drug demonstrates potential efficacy in treating both nociceptive and neuropathic pain. Preclinical and early clinical studies have shown effective inhibition of pain signal conduction by sensory fibers, without affecting motor functions or other sodium channels not involved in pain perception [8].

Initial investigations into NaV1.7 inhibitors did not yield the expected clinical outcomes due to insufficient spinal inhibition, short duration of action, toxicity, and limited bioavailability. Development of NaV1.9 inhibitors faced challenges related to the expression of functional channels in vitro, redirecting focus to NaV1.8.

**Figure 2:**
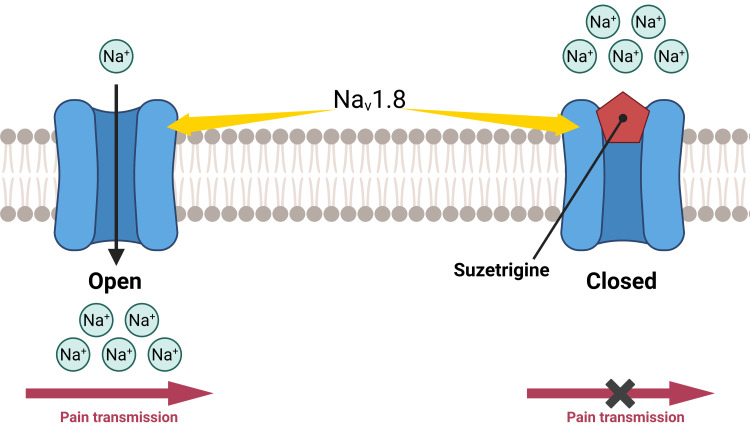
Suzetrigine's mechanism of action Created under a Creative Commons license in BioRender (https://BioRender.com/6qh0nzb) Image credit: Maciej Mach

The voltage-gated sodium channel NaV1.8 plays a critical role in nociceptive signal transmission and represents an attractive therapeutic target for pain management (Figure 2). Its selective expression in peripheral nociceptive neurons of the dorsal root ganglia, and its involvement in physiological sensory responses, underscore its importance in pain conduction mechanisms.

The role of NaV1.8 has been confirmed in studies of SCN10A gene mutations, animal models, and pharmacological analyses of agents modulating its activity. NaV1.8 plays a key role in the generation and propagation of action potentials in pain pathways, particularly under conditions of inflammation or nerve injury [9].

By selectively blocking NaV1.8, suzetrigine reduces neuronal hyperexcitability without affecting central nervous system function, thereby providing analgesia with a lower risk of addiction or sedation compared to opioids. Its peripherally restricted action minimizes central side effects, making it a promising non-opioid treatment for moderate to severe acute pain [5,9]. This mechanism is especially relevant, as it targets pathological pain signaling at its origin - within peripheral sensory neurons - rather than centrally modulating pain perception.

Furthermore, suzetrigine is a highly potent inhibitor of the NaV1.8 channel, demonstrating exceptional selectivity, with a half-maximal inhibitory concentration (IC_50_) of 0.27 nanomoles (nM) and over 30,000-fold preference compared to other sodium channel subtypes. At increased concentrations (IC_50_ of 200 nM), it also shows efficacy in blocking sodium channels in an in vivo animal model [10].

Studies have demonstrated that orally administered suzetrigine interacts with NaV1.8 by stabilizing the channel in a closed state. Confirmation of this mechanism requires intense and prolonged depolarization of cells to reveal the effect of relief from inhibition, described as reverse use-dependence [11].

Although, theoretically, this could reduce efficacy under conditions of repetitive stimulation, results indicate that suzetrigine effectively maintains inhibition even during repeated depolarizations mimicking nociceptive signals in sensory neurons. Thus, it can be classified as a tonic NaV1.8 inhibitor, as its activity is not dependent on channel opening and persists across a wide range of voltages and impulse frequencies characteristic of painful states [8].

However, modulation of NaV channels must be approached with caution due to their expression in cardiomyocytes and potential cardiovascular effects. NaV1.8 mutations have been linked to Brugada syndrome, and their overexpression may contribute to heart failure and cardiac hypertrophy.

Lidocaine, a non-selective NaV inhibitor used in pain treatment, exhibits analgesic effects at low doses, but higher doses increase the risk of cardiac complications due to its impact on other NaV subtypes, such as NaV1.5, which is essential for cardiac function [6].

Pharmacokinetics

Suzetrigine is well absorbed following oral administration and exhibits a long half-life of approximately 30-60 hours, enabling once- or twice-daily dosing while maintaining plasma concentrations in the low-nanomolar range required for near-complete inhibition of NaV1.8 (IC_50_ in humans ≈ 0.68 nM) in clinical studies [8,12]. It is primarily metabolized in the liver via glucuronidation, with minimal renal excretion; thus, caution is recommended in patients with hepatic impairment, whereas dose adjustment is generally unnecessary in those with renal dysfunction [8,12].

Preclinical studies have shown that its pharmacokinetics may vary significantly by sex, with female rats exhibiting markedly higher systemic exposure, area under the curve (AUC_0-t_) up to 50-fold greater, and oral bioavailability (96% vs. 11% in males), due to slower metabolism via cytochrome (CYP) CYP3A2- and CYP2C11-mediated demethylation [13].

Animal pharmacokinetic profiling in monkeys confirmed good oral bioavailability and stable plasma levels, with validated liquid chromatography-tandem mass spectrometry (LC-MS/MS) methods supporting accurate quantification in preclinical trials [14].

Distribution studies show that suzetrigine selectively acts on peripheral sensory neurons, with no detectable SCN10A (NaV1.8 gene) expression in the central nervous system [15]. This peripherally restricted activity reduces the likelihood of central nervous system side effects.

Across species, potency varies, with the highest sensitivity observed in humans and non-human primates. Its low potential for drug-drug interactions, particularly with other analgesic drugs, makes it an attractive candidate for combination therapy [4]. However, the prolonged half-life may delay steady-state attainment and extend the duration of adverse effects, necessitating careful dose titration [8,16].

Indications

Effective management of acute pain requires a balance between providing adequate analgesia and minimizing the risk of adverse effects [17]. Poorly controlled pain can lead to complications such as delayed recovery, prolonged hospital stays, and reduced quality of life [18]. Furthermore, inadequate treatment of acute pain increases the risk of its transition to a chronic form, which carries significant socio-economic costs [19,20].

Currently, the treatment of acute pain involves a multimodal strategy, incorporating different classes of medications, including NSAIDs, acetaminophen, local anesthetics, and NMDA receptor antagonists [3]. Despite the availability of the above-mentioned options, opioids remain a cornerstone in the treatment of moderate to severe pain. However, their use is associated with adverse effects such as sedation, respiratory depression, nausea, vomiting, constipation, and renal dysfunction [21].

Moreover, opioid pain treatment in the USA leads to approximately 85,000 cases of opioid use disorder (OUD) annually [22,23]. Managing pain in such individuals remains a clinical challenge due to the risk of relapse and the limited range of effective non-opioid alternatives [22,23].

In this context, suzetrigine has been investigated as a promising solution, as preclinical and early clinical studies suggest that it exerts analgesic effects through selective inhibition of peripheral sodium channels, without engaging opioid receptors [5,9]. This mechanism is hypothesized to lower the risk of abuse or physical dependence compared with opioids, which could make suzetrigine a potentially valuable option for patients with a history of substance use disorders [24]. Selective inhibition of the voltage-gated sodium channel NaV1.8 has emerged as a promising therapeutic approach, with NaV1.8 inhibitors showing potential to provide effective and well-tolerated analgesia in acute and chronic pain [25,26].

Suzetrigine has the potential to alleviate both acute postoperative pain and chronic neuropathic pain - including pain associated with neuralgia, diabetes, or peripheral nerve injury. Given its mechanism of action and safety profile, it may be suitable for patients with moderate to severe pain, particularly when other treatments are ineffective, poorly tolerated, or when opioid use is contraindicated or carries a risk of dependence [24,27-29].

Blocking NaV1.8 channels allows for selective pain suppression without a generalized depressive effect on the central nervous system, distinguishing suzetrigine from opioids and making it a novel alternative in pain management [8]. Nevertheless, further large-scale clinical studies are needed to confirm its efficacy and safety. Data on its use in multimodal analgesia, and in pregnant or breastfeeding patients, are also of particular importance [6].

Efficacy

NaV1.8 channels represent an attractive therapeutic target for pain management due to their predominant expression in primary sensory neurons and minimal presence in most other neuron types [11]. Suzetrigine has demonstrated high efficacy in pain reduction during clinical trials and has shown greater potency compared to another compound with a similar mechanism of action, VX-150. It exhibits a unique “reverse use-dependence,” meaning its inhibitory effect can be attenuated by repetitive depolarizations [11]. This process occurs with a time constant of approximately 40 milliseconds (ms), independent of drug concentration, whereas re-inhibition at negative voltages is proportional to concentration. This suggests that suzetrigine binds strongly to NaV1.8 channels in the resting state, but much less so to channels with fully activated voltage sensors, indicating a distinctive state-dependent inhibition [11].

Two pivotal randomized controlled trials (NAVIGATE-1 and NAVIGATE-2) assessed the efficacy of suzetrigine in adult patients experiencing moderate to severe acute pain following abdominoplasty and bunionectomy [30]. Patients receiving suzetrigine - a 100-milligram (mg) loading dose, followed by 50 mg every 12 hours - reported significantly greater pain relief compared to placebo, as measured by the sum of pain intensity differences over 48 hours (SPID48) outcome [30]. In the abdominoplasty trial, the least squares mean difference (LSMD) between suzetrigine and placebo was 48.4 (p < 0.0001), while in the bunionectomy trial it was 29.3 (p = 0.0002) [30]. Suzetrigine also reduced pain more rapidly than placebo, with a clinically meaningful numeric pain rating scale (NPRS) decrease achieved in two to four hours versus eight hours with placebo [30]. Compared to low-dose opioids - hydrocodone/acetaminophen (HB5/APAP325) - suzetrigine showed similar or slightly lower efficacy, depending on the procedure: it performed better in abdominoplasty but was inferior in bunionectomy [30]. A network meta-analysis further indicated that suzetrigine’s analgesic effect was comparable to that of NSAIDs and slightly lower than high-dose opioids [30]. Overall, suzetrigine was well tolerated, with fewer gastrointestinal side effects than opioids, and a discontinuation rate below 1% [30].

In a single-arm study assessing suzetrigine’s effectiveness in treating acute pain of both surgical and non-surgical origin, 83.2% of patients experiencing moderate to severe pain rated the treatment as good, very good, or excellent on the Patient Global Assessment (PGA) scale [31]. In this study, participants were allowed to take additional acetaminophen (650 mg) and ibuprofen (400 mg) every six hours, referred to as rescue medication, whenever extra pain relief was necessary. Notably, the high rate of positive assessments was consistent regardless of pain type - 82.0% among surgical patients and 91.2% among non-surgical patients - and suzetrigine’s efficacy was not significantly influenced by the use of rescue medications [31]. Only 1.6% of patients discontinued therapy due to lack of efficacy, further confirming the drug’s favorable tolerability profile [31].

Dosage

Phase II and III clinical trials evaluated the efficacy and safety of various dosing regimens for the treatment of acute postoperative pain [5,6,32]. Both phases employed the same regimen: a loading dose of 100 mg, followed by 50 mg every 12 hours for 48 hours post-surgery.

In two Phase II studies involving patients after abdominoplasty and hallux valgus correction surgery, a significant reduction in pain intensity (SPID) over 48 hours was observed only in the high-dose group compared to placebo [5]. Lower doses did not demonstrate significant superiority over either placebo or hydrocodone combined with acetaminophen. These findings were confirmed in two Phase III trials including over 2,000 patients - the high-dose regimen provided a rapid and sustained analgesic effect clearly superior to placebo, though comparable or slightly less potent than hydrocodone with acetaminophen [32]. The 100 mg loading dose, followed by 50 mg every 12 hours, is currently considered the most effective dosing regimen among those studied to date [6].

Adverse effects

Preclinical studies in rats and monkeys showed no significant adverse effects or potential for dependence [8]. No neurobehavioral, sedative, or stimulatory effects were observed. Suzetrigine did not induce physical dependence symptoms after abrupt discontinuation, even at doses many times higher than therapeutic levels. No adverse effects on the cardiovascular or respiratory systems - including blood pressure or electrocardiogram (ECG) changes - were reported, even with long-term use.

In two Phase II clinical trials involving patients with acute pain following abdominoplasty (n = 303) and hallux valgus surgery (n = 274), the drug demonstrated good tolerability and a favorable safety profile [5]. The most common adverse events (≥10%) were nausea, headache, constipation, dizziness, and vomiting. These symptoms were mild, resolved spontaneously or with symptomatic treatment, and did not lead to treatment discontinuation. Serious adverse events - such as pulmonary embolism (in the group receiving an average dose of suzetrigine), laryngeal stenosis (hydrocodone with acetaminophen group), and cellulitis with sepsis (placebo group) - occurred in isolated patients and were not related to the study drug. Treatment discontinuation rates were lower in suzetrigine groups compared to placebo and hydrocodone/acetaminophen groups.

A separate clinical safety study confirmed good drug tolerability. Most adverse events were mild or moderate, with the most frequent being headache, constipation, nausea, falls, and rash; only headache occurred in more than 5% of participants (7.0%) [28]. Serious adverse events were reported in only two patients (0.8%) - one with suicidal ideation and another who developed cellulitis. Both cases were deemed unrelated to suzetrigine therapy [31].

Some patients also experienced skin rash and mild cognitive symptoms, such as difficulty concentrating. These reactions appear dose-dependent and are generally mild to moderate in severity [8]. Despite initial optimism, concerns arose regarding potential neurotoxicity at higher doses and proarrhythmic risk observed in in vitro studies [8]. Additionally, like other sodium channel blockers, suzetrigine may carry a risk of hypersensitivity reactions, although this appears less frequent than with lamotrigine. Due to hepatic metabolism, the drug should be used cautiously - or avoided - in patients with severe liver impairment [8]. There are no data on suzetrigine use during pregnancy, and teratogenic risk cannot be excluded; therefore, the drug is contraindicated in pregnant women until further safety data are available.

Drug-drug interactions

Suzetrigine is both a substrate and an inducer of CYP3A4, which can lead to significant drug interactions [33,34]. Concomitant use with strong CYP3A4 inhibitors, such as ketoconazole, is contraindicated, while dose adjustment is recommended for moderate CYP3A4 inhibitors. Products containing grapefruit should also be avoided due to CYP3A4 inhibition [33,34]. Suzetrigine may reduce the efficacy of sensitive CYP3A4 substrates, including midazolam, and requires careful management of hormonal contraceptives other than levonorgestrel and norethindrone, with a backup nonhormonal method recommended during treatment and for 28 days after discontinuation [33,34]. The impact of suzetrigine on CYP3A4 substrates used in OUD therapy, such as buprenorphine and methadone, remains to be fully established [34]. More pharmacokinetic studies are currently underway to evaluate potential interactions between suzetrigine and other therapeutic agents, including the effects of concomitant use of CYP450 enzyme inhibitors or inducers on the metabolism and bioavailability of the drug [6].

Cost-effectivness

A two-part economic model assessed the cost-effectiveness of suzetrigine versus HB/APAP for treating moderate to severe acute pain, including a short-term decision tree (for the initial seven-day treatment) and a long-term Markov model capturing lifetime outcomes related to OUD [24]. Four health states were modeled: no OUD, OUD, abstinence, and death, with OUD associated with significantly worse quality of life, survival, and healthcare costs. The analysis assumed that suzetrigine carries zero risk of inducing OUD, while 0.43% of patients treated with HB/APAP would develop OUD within three years [24]. Based on wholesale pricing, total discounted lifetime costs for suzetrigine were about $400 lower than for HB/APAP, while yielding slightly higher quality-adjusted life years (QALYs) and life expectancy [24]. Suzetrigine also led to 429 fewer OUD cases per 100,000 patients [24]. Suzetrigine offers faster pain relief in certain procedures, fewer adverse events, and a lower projected risk of OUD than HB/APAP, potentially improving both patient adherence and quality of life [24]. These findings indicate that suzetrigine is a dominant strategy - both less expensive and more effective - compared to opioid-based therapy. The cost-effectiveness was especially sensitive to OUD-related risks and costs associated with HB/APAP [24]. The article’s key limitations relate to uncertainty in the cost-effectiveness modeling, particularly regarding the risk of OUD following short-term use of HB/APAP compared with suzetrigine [24]. The actual incidence of OUD after acute pain treatment is unclear, and the model relies on assumptions that may underestimate or overestimate this risk [35]. Additionally, there are limited data on suzetrigine’s potential to cause OUD, making its long-term safety uncertain. 

Future research should focus on better understanding the risk of OUD associated with both acute and chronic pain treatments, distinguishing between opioid and non-opioid therapies [35]. Specifically, studies are needed to clarify suzetrigine’s potential risk for OUD, and to provide more precise estimates of OUD incidence following short-term HB/APAP use. Such research would help refine cost-effectiveness models and ensure that assumptions about OUD risks accurately reflect real-world outcomes.

Advantages of suzetrigine over existing pain medications

Suzetrigine offers an alternative to traditional opioids for managing moderate-to-severe acute pain by providing rapid, clinically meaningful analgesia while avoiding the sedation, euphoria, and addictive potential associated with opioid therapy [22,31]. Clinical trials in postoperative settings such as abdominoplasty and bunionectomy have demonstrated significant reductions in pain intensity (SPID48 up to 48.4%, p < 0.001), with a median onset of relief in just two hours, comparable to hydrocodone/acetaminophen but with better tolerability and fewer adverse events [22,36]. Unlike NSAIDs or opioids, suzetrigine’s targeted peripheral action selectively inhibits NaV1.8 channels in dorsal root ganglia neurons, preventing pain signal transmission without central nervous system side effects, making it particularly suitable for patients at high risk of opioid dependence or adverse drug reactions [31,35]. Furthermore, its favorable safety profile, absence of abuse potential, and potential role in opioid-sparing regimens position suzetrigine as a significant advancement in acute pain management, addressing the urgent need for effective, non-addictive analgesics in the context of the ongoing opioid crisis [31,36,37].

## Conclusions

Suzetrigine is a highly selective oral NaV1.8 sodium channel inhibitor that represents a significant advancement in the management of moderate to severe acute pain. Its peripheral, non-opioid mechanism of action allows for effective analgesia without engaging central opioid receptors, thus eliminating risks such as respiratory depression, sedation, and OUD. Clinical trials have demonstrated its efficacy in both surgical and non-surgical pain, with a favorable safety and tolerability profile. The drug showed comparable effectiveness to NSAIDs and low-dose opioids, and, while slightly less potent than high-dose opioids in some settings, it was associated with fewer gastrointestinal and central nervous system side effects. Adverse events were typically mild to moderate, and the incidence of serious drug-related complications was low. Pharmacokinetic properties, including a long half-life and hepatic metabolism, support convenient dosing but require caution in patients with liver impairment. Cost-effectiveness modeling suggests that suzetrigine not only reduces the burden of opioid-related complications, such as addiction, but may also offer long-term economic advantages. While ongoing studies are needed to further clarify long-term safety, drug interactions, and efficacy in special populations, current evidence supports suzetrigine as a promising, safe, and effective non-opioid alternative in acute pain management - especially valuable in the context of the ongoing opioid crisis.
